# Effectiveness of mHealth Nutrition Interventions on Nutrition-Related and Clinical Outcomes in Adults with Chronic Kidney Disease: A Systematic Review and Meta-Analysis

**DOI:** 10.3390/nu18203357

**Published:** 2026-10-13

**Authors:** Samantha Walter-Cano, Allison Lee, Jeanette M. Andrade

**Affiliations:** Food Science and Human Nutrition Department, University of Florida, Gainesville, FL 32611, USA; walter-cano.s@ufl.edu (S.W.-C.); all5@ufl.edu (A.L.)

**Keywords:** mHealth, chronic kidney disease, nutrition-related outcomes, clinical outcomes, systematic review and meta-analysis

## Abstract

Background/Objectives: Diet recommendations for individuals with chronic kidney disease (CKD) are complex and can be challenging to integrate into daily routines. Mobile health (mHealth) interventions may support dietary education, self-monitoring, and disease management; however, their effects on nutrition-related and clinical outcomes in CKD remain unclear. This systematic review and meta-analysis evaluated the effectiveness of mHealth interventions on nutrition-related and clinical outcomes among adults with CKD. Methods: The review was conducted according to the Preferred Reporting Items for Systematic Review and Meta-analysis (PRISMA) guidelines. Five databases were searched from inception through February 2026. Controlled trials, quasi-experimental designs and pre–post studies evaluating mHealth interventions and reporting nutrition-related and/or clinical outcomes were eligible for inclusion. Randomized controlled trials and controlled quasi-experimental studies with comparable outcomes and sufficient data were included in the quantitative synthesis. Pre–post studies were synthesized narratively because changes from baseline could not be distinguished from temporal effects in the absence of a concurrent comparator. Risk of bias was assessed using the Cochrane Risk of Bias tool and ROBINS-I. Results: Twenty-three studies were included in the review, with eight meeting the inclusion criteria for a meta-analysis. Among the quantitative synthesis of adults on dialysis, no significant effects were shown with biochemical outcomes or interdialytic weight gain. Several pre–post studies reported within-study improvements in dietary intake, including sodium intake, and blood pressure outcomes. These findings should be interpreted cautiously because the absence of a concurrent control group precludes attributing observed changes specifically to the mHealth intervention. Conclusions: mHealth interventions may support improvements in selected dietary and clinical outcomes among adults with CKD; however, evidence from randomized controlled trials remains limited. Improvements reported in pre/post studies should be interpreted cautiously and additional well-designed randomized controlled trials are needed to determine their effectiveness across CKD populations.

## 1. Introduction

Chronic kidney disease (CKD) is a progressive disease that has emerged as a major global public health issue, with the prevalence more than doubling between 1990 and 2023 [1,2]. As kidney function declines, the kidney’s ability to eliminate metabolic waste products and regulate fluid and electrolyte balance diminishes. Although nonmodifiable factors such as genetics and age contribute to CKD progression, modifiable lifestyle factors such as diet are an important component for CKD management.

Healthcare providers routinely counsel individuals with CKD on dietary modifications tailored to their individual health conditions [3,4]. This process typically begins with providers and registered dietitian nutritionists (RDNs) identifying dietary goals based on the individual’s medical condition, followed by translating these recommendations into practical food choices, meal planning strategies, and educational resources. These individuals are then encouraged to incorporate these recommendations into their daily routines with support from an interdisciplinary healthcare team, including physicians, nurses, RDNs, and other allied health professionals [3,4]. However, translating nutrition recommendations into sustained dietary behaviors remains challenging [5]. Individuals must navigate competing dietary recommendations for multiple chronic conditions, financial constraints, limited access to healthy foods, cultural food preferences, time required for meal preparation, and varying levels of food and nutrition literacy [5,6,7]. This gap between evidence-based recommendations and real-world adherence complicates effective dietary management of CKD, highlighting the need for innovative strategies to support self-management. Thus, digital health interventions may offer new and enhanced strategies for dietary monitoring and adherence in this population.

Among digital health interventions, mobile health (mHealth) has gained increasing attention as a tool to facilitate self-management and improve chronic disease outcomes. Various definitions of mHealth have been proposed; however, the rapid evolution of mobile technology has made establishing a universal definition challenging [8]. The World Health Organization (WHO), for example, broadly defines the term as a medical and public health practice supported by mobile and wireless devices. These devices include examples such as smartphones, sensors, personal digital assistants (PDAs), and wireless monitoring devices, among others [9]. However, modern interventions often push past these traditional boundaries. Earlier mHealth interventions frequently relied on technologies such as PDAs and short message service (SMS), but advances in mobile technology have expanded the capabilities of these interventions. Contemporary mHealth approaches may incorporate real-time monitoring, wearable device integration, personalized feedback, remote communication with healthcare providers, and, more recently, artificial intelligence (AI)-supported features [10,11]. These advances have expanded the potential for mHealth to provide increasingly individualized support for self-management without requiring continuous interaction with a healthcare provider.

Recent attention in mHealth interventions has largely focused on non-communicable diseases, particularly type 2 diabetes. Recent data from a 2025 systematic review that focused on individuals with type 2 diabetes demonstrated that SMS-based mHealth interventions improved glycemic control, with most of the studies reporting significant reductions in hemoglobin A1c (HbA1c) compared with standard care [12]. Another recent study on individuals with uncontrolled type 2 diabetes and high cardiovascular risk showed significant improvements in both HbA1c levels and systolic blood pressure (SBP) using a message-based mHealth intervention [13]. While these findings suggest that mHealth technologies can effectively support chronic disease self-management and improve clinical outcomes, the evidence supporting the use of these mHealth interventions within the CKD population remains limited. Additionally, mHealth is frequently incorporated as one component of multicomponent interventions that also include counseling or education, making it difficult to isolate the effects attributable specifically to mHealth. A prior systematic review published in 2015 evaluated dietary mobile apps in individuals with CKD and suggested potential clinical benefits; however, there were few included studies and they varied in design, length, intervention, and nutritional outcome assessments [14]. Despite these advancements, recent systematic reviews and meta-analyses evaluating the effectiveness of mHealth interventions within the CKD population remain limited. While meta-analyses regarding mHealth in other chronic diseases suggest considerable improvements in clinical and dietary outcomes with mHealth interventions, it remains unclear whether similar benefits extend to the CKD population. Therefore, the purpose of this systematic review and meta-analysis is to determine the effectiveness of mHealth interventions on nutrition-related and clinical outcomes among adults with CKD.

## 2. Materials and Methods

### 2.1. Selection of the Studies

This systematic review and meta-analysis were conducted in accordance with the Preferred Reporting Items for Systematic Reviews and Meta-Analyses (PRISMA) [15]. The study protocol was registered in the prospective register of systematic reviews database (registration code: CRD420251126284). Potentially relevant published peer-reviewed journal articles were initially identified based on nested keyword searches. This was then followed by reviewing the title and abstracts of the articles based on determined inclusion/exclusion criteria. Finally, an assessment of the relevant articles that were closely associated with the original intent of the search was performed. Five electronic databases, Pubmed/Medline, Embase, Cochrane Library, Scielo, and Scopus, were searched from inception through September 2025. The searches were updated in February 2026 using the same search strategy across all databases to identify any additional eligible studies published since the initial search.

**Figure 1:**
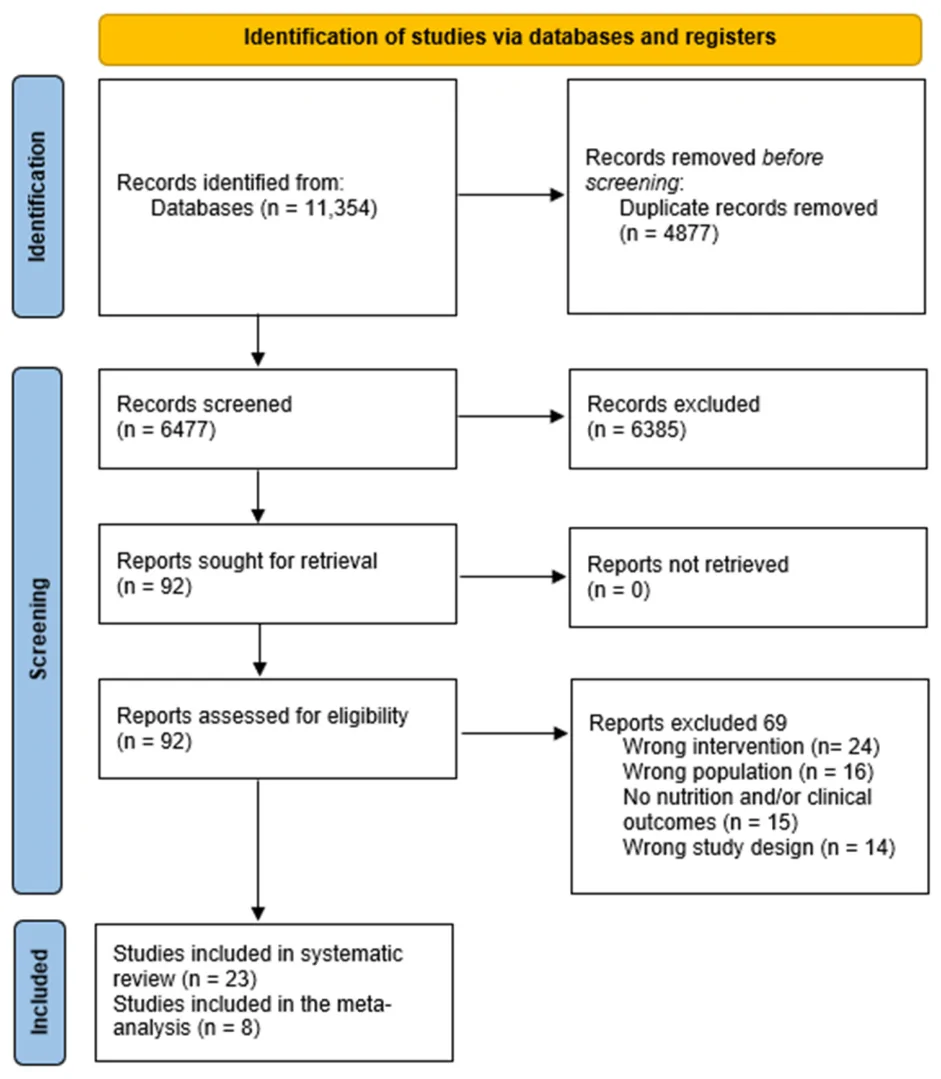
PRISMA flow diagram.

Two researchers (AL and SWC) conducted a comprehensive literature search that incorporated terms related to kidney disease, mHealth technologies, and nutrition-related outcomes. Kidney-related terms included chronic kidney disease, kidney or renal disease, failure, insufficiency, function, and dysfunction. mHealth- and technology-related terms included mobile health (mHealth), applications/apps, smartphones, wearable devices, monitoring platforms, and other wireless technologies. Some included interventions incorporated text messaging or SMS as a communication or support component within a broader mHealth intervention rather than as the primary intervention modality. Nutrition-related search terms included phosphorus, potassium, sodium, albumin, total protein, and dietary intake or habits. Search terms were adapted as appropriate for each database. The complete search strategy is provided in Supplemental Table S1. Given that no human or animal subjects were involved, institutional review board (IRB) approval was not required. The search was completed for peer-reviewed articles written in English to encompass the vast literature available. Search results were imported into Covidence (Veritas Health Innovation, Melbourne, Australia) for duplicate removal and study screening. Initially, 11,354 articles were identified for a total of 23 articles included in the review and eight for the meta-analysis. Any discrepancies were discussed with the research team. See Figure 1 for a flow diagram of the article selection process.

**Table 1:** mHealth studies (*n* = 23).

Authors, Date of Publication	Country	Population	Design	Length (in Months)	mHealth Platform	Nutrition Intervention Components	Comparator	Biochemical Outcomes	Behavioral Outcomes	Clinical Outcomes
Cao et al., 2018 [16]	China	PD	RCT	12	QQ instant messaging	Nutrition education; reminders; clinician communication	Standard care	↑ Albumin; ↓ P; ↔ Ca	↑ Patient satisfaction	NR
Chae et al., 2024 ^+^ [17]	South Korea	PD	RCT	2.3	Mobile app ^A^	CKD education; self-monitoring; personal tracking; reminders	Standard care	↑ Albumin; ↑ Hemoglobin; ↔ P; ↔ K	↑ CKD Knowledge; ↑ Health behaviors; ↑ HRQoL; ↔ Self-efficacy	NR
Chang et al., 2020 ^+^ [18]	United States	CKD stages 1–3	Pre/post	1.8	Mobile app	Dietary intake; weekly calls	None	↔ Albuminuria; ↔ Urine Na	↓ Dietary Na; ↔ Diet quality	↔ SBP; ↓ DBP
Chiang et al., 2021 [19]	Taiwan	HD	Quasi-experimental	0.5	Mobile app ^A^	Food/phosphate tracking; personalized dietary feedback	Standard care	↔ Albumin; ↔ P	↑ P knowledge; ↑ Self-efficacy	NR
Dawson et al., 2021 ^+^ [20]	Australia	HD	RCT	6	Text messaging ^A^	Nutrition education; motivational support	Standard care	↔ Albumin; ↔ P; ↔ K	↓ Dietary protein; ↓ Dietary P; ↑ QoL	↓ IDWG
Doyle et al., 2019 [21]	Ireland	CKD stages 2–5	Pre/post	2.8	Mobile app ^A^	MiKidney app; diet recording; symptom management	None	NR	↑ Diet intake	↔ weight; ↓ waist circumference
El Khoury et al., 2021 ^+^ [22]	United Arab Emirates	HD	Pre/post	0.5	Mobile app ^A^	Dietary education; renal recipes	None	↔ P	↑ Knowledge; ↔ Adherence to app	NR
Farfan-Ruiz et al., 2021 [23]	Canada	PD	RCT	2.8	Mobile app ^A^	Phosphate counting; meal phosphate calculation; binder recommendations	Standard care	↔ Ca; ↔ P	↔ Technology readiness	NR
Imtiaz et al., 2017 [24]	Canada	PD	Pre/post	0.9	Mobile app ^A^	Phosphate counting; meal phosphate calculation; binder recommendations	None	↔ Ca; ↔ P	Technology readiness: descriptive data only	None
Jin et al., 2024 [25]	China	HD	Pre/post	0.9	GPT tool	Meal-photo analysis; real-time K feedback	None	↓ K	NR	NR
Jin et al., 2024 [26]	China	PD	Pre/post	0.9	GPT tool	Personalized renal recipes; nutrition guidance	Standard care	↑ Prealbumin; ↔ Albumin; ↔ P	NR	NR
Jung et al., 2023 [27]	South Korea	CKD stages 1–5	Pre/post	6	Mobile app	Diet intake	None	↓ Urine Na	↓ diet Na intake	NR
Kim & Cho, 2025 ^+^ [28]	South Korea	HD	RCT	0.9	Mobile app ^A^	Individualized feedback; online community	Standard care	↔ P; ↔ K	↑ Self-efficacy; ↑ Self-care	↓ IDWG
Lee & Kang, 2024 ^+^ [29]	South Korea	PD	Quasi-experimental	1.2	Mobile messaging ^A^	Customized diet education; nurse interaction	Standard care	↔ Albumin; ↔ P; ↔ K	↑ Dietary compliance; ↑ Self-efficacy	NR
Ong et al., 2016 [30]	Canada	CKD stages 4–5	Pre/post	6	Mobile app ^A^	Self-management; personalized feedback	None	↔ Ca; ↔ P	NR	↓ SBP; ↓ DBP
Pinto et al., 2020 [31]	Brazil	HD	Pre/post	4	Mobile app ^A^	Diet/fluid monitoring; nutrient guidance	Self-control	↓ P; ↔ Ca; ↔ K	↑ Self-management	↔ IDWG
Schrauben et al., 2022 ^+^ [32]	United States	CKD stages 1–3	Pre/post	12	Mobile app	Tele-counseling; emails; self-monitoring diet	None	↔ Urine Na; ↓ Urine K; ↓ Urine P	↑ Protein intake; ↑ Diet quality	↓ SBP; ↓ DBP
Sevick et al., 2005 ^+^ [33]	United States	HD	Quasi-experimental	3.7	PDA-based Mobile app ^A^	PDA-based diet tracking; dietary self-monitoring	None	Albumin: descriptive data only; P: descriptive data only	None	IDWG: descriptive data only
Shi et al., 2024 ^+^ [34]	China	CKD stages 1–5	Retrospective cohort	36	Mobile app ^A^	Self-management; education, personalized feedback	Standard care	↔ Albumin; ↓ Total protein; ↔ Ca; ↔ P; ↓ Na	NR	NR
St-Jules et al., 2023 ^+^ [35]	United States	CKD stages 1–3	RCT	6	Mobile app	Dietary/physical activity monitoring; behavioral counseling	Advice-written information	↔ Urine Na:Cr; ↔ Urine P:Cr	None	None
Torabikhah et al., 2023 [36]	Iran	HD	RCT	1.4	Mobile app ^A^	Diet/fluid education; HD self-management education	Face-to-face education	↔ Albumin; ↔ P; ↔ K	NR	↓ IDWG
Waki et al., 2024 ^+^ [37]	Japan	CKD stages 1–3	RCT	12	Mobile app ^A^	Exercise, blood glucose, diet, blood pressure, and body weight monitoring.	Standard Care	↓ Albuminuria	None	↔ SBP; ↔ DBP
Welch et al., 2013 ^+^ [38]	United States	HD	RCT	3.7	Mobile app ^A^	Dietary/fluid self-monitoring; real-time feedback	Paper diary	NR	↑ Perceived control; ↔ dietary outcomes	↔ IDWG

**Note.** mHealth platform = digital technology used to deliver the intervention; nutrition intervention components = behavior-change strategies delivered through the platform. + indicates intervention development was informed by one or more behavioral theories, models and/or frameworks. ^A^ indicates studies that developed their own application. Unless otherwise specified as urinary, biochemical markers refer to serum concentrations. ↑ = statistically significant increase; ↓ = statistically significant decrease; ↔ = no statistically significant difference. Arrows represent changes or differences as reported by the original study according to its study design and analysis. Descriptive data only = outcome was reported without inferential statistical testing. None = outcome or component was not included or assessed; NR = not reported. Na:Cr = sodium-to-creatinine ratio; P:Cr = phosphorus-to-creatinine ratio; BP = blood pressure; Ca = calcium; CKD = chronic kidney disease; Cr = creatinine; DBP = diastolic blood pressure; GPT = generative pretrained transformer; HD = hemodialysis; HRQoL = health-related quality of life; IDWG = interdialytic weight gain; K = potassium; Na = sodium; P = phosphorus; PD = peritoneal dialysis; PDA = personal digital assistant; QoL = quality of life; RCT = randomized controlled trial; SBP = systolic blood pressure; SCT = social cognitive theory.

### 2.2. Eligibility Criteria

Studies were included if they met the following criteria: (1) study design was considered randomized controlled trial (RCT), quasi-experimental, or pre/post; (2) included participants aged 18 years or older with CKD of all stages (1–5, including dialysis); (3) interventions involved mobile health (mHealth) technologies (e.g., apps, smartphones, wearables, other wireless devices); and (4) reported nutritional outcomes (e.g., electrolytes, albumin, total protein, and/or dietary intake). Studies were excluded if they did not include the above criteria and involved specific kidney conditions such as autoimmune disease (e.g., lupus, IgA nephropathy), polycystic kidney disease, Mesoamerican nephropathy, urinary tract problems, or acute renal failure; included pregnant women or children; evaluated telehealth or online counseling services without an mHealth component; did not include nutritional outcomes; focused on the development or design of mHealth; and/or were not full-text articles published in non-scientific journals (e.g., conference abstracts, books, ongoing studies).

### 2.3. Data Extraction

All titles and abstracts were screened independently by three reviewers (JMA, AL, and SWC), and full-text studies that were considered relevant were included for further review. The reviewers independently reviewed all full-text articles, and any discrepancies were resolved with consensus. Data from the studies that fulfilled the eligibility criterion were collected in Covidence: (1) title, (2) author’s last name, (3) year of publication, (4) location of study, (5) study design, (6) population type and number, (7) length of intervention, (8) intervention and control, (9) outcomes, and (10) results.

### 2.4. Quality and Risk of Bias Assessment

Risk of bias for randomized controlled trials was assessed using the original Cochrane Risk of Bias tool (RoB 1), which evaluates seven domains [39]. Seven total domains were used to identify risk of bias. The domains assessed were sequence generation, allocation of concealment, blinding of participants and personnel, blinding of outcome assessors, incomplete outcome data, selective outcome reporting, and other sources of bias. These domains were assessed by indicating either a high, low, or unclear bias; with an “unclear” bias indicating that insufficient information was available to determine whether the domain was addressed.

Assessment of non-RCTs was done by using the ROBINS-I tool [40]. There are seven domains that were assessed: bias due to confounding, selection of participants, classification of the intervention, deviations from intended interventions, missing outcome data, measurement of outcomes, and selection of the reported result. Domain-level judgments were categorized as low, moderate, serious, critical risk of bias, or no information. An overall risk-of-bias judgment was determined according to the ROBINS-I framework based on the domain-level assessments. Risk-of-bias visualizations were generated using the robvis package [41].

### 2.5. Assessment of Heterogeneity

Heterogeneity was assessed using the I^2^ statistic. Publication bias was explored through visual inspection of funnel plots; however, because of the small number of studies available for most outcomes, funnel plots were interpreted cautiously. Statistical heterogeneity was determined at a chi-square (*p* < 0.01) and an I^2^ value of at least 50%.

### 2.6. Data Synthesis

Studies were grouped according to treatment modality (dialysis or not), intervention and comparator characteristics, and nutrition-related or clinical outcomes. Randomized controlled trials and controlled quasi-experimental studies were eligible for quantitative synthesis when they evaluated comparable populations and outcomes and provided sufficient data to estimate an intervention effect. Uncontrolled pre–post studies were excluded from the quantitative synthesis and were summarized narratively. For each outcome, quantitative synthesis was limited to studies reporting comparable intervention and control group data with sufficient information to calculate an effect estimate and its variance; studies not meeting these criteria were summarized narratively. For the studies that involved hemo- and peritoneal dialysis populations, they were grouped together when the intervention, comparator, and outcome measures were considered clinically comparable. Modality-specific outcomes were analyzed separately. Data from each included trial were analyzed using Review Manager (RevMan, Version 7.2; The Cochrane Collaboration, Copenhagen, Denmark). Treatment effects were expressed as between-group mean differences in final follow-up values with corresponding 95% confidence intervals (CIs). When standard deviations were not directly reported, they were estimated from available summary statistics, including 95% confidence intervals, using methods described in the Cochrane Handbook. For one study, standard deviations were approximated from rounded, model-based confidence intervals; therefore, these estimates were considered subject to additional uncertainty. A random-effect model was applied to determine differences. Statistical significance was defined as *p* < 0.05.

## 3. Results

### 3.1. Study Characteristics

Twenty-three studies met the inclusion criteria and were conducted in Australia (*n* = 1) [20], Brazil (*n* = 1) [31], Canada (*n* = 3) [23,24,30], China (*n* = 4) [16,25,26,34], Iran (*n* = 1) [36], Ireland (*n* = 1) [21], Japan (*n* = 1) [37], South Korea (*n* = 4) [17,27,28,29], Taiwan (*n* = 1) [19], United Arab Emirates (*n* = 1) [22], and the United States (*n* = 5) [18,32,33,35,38,42]. Study designs included randomized controlled trials (RCTs; *n* = 9) [16,17,20,23,28,35,36,37,38], quasi-experimental studies (*n* = 3) [19,29,33], a retrospective cohort study (*n* = 1) [34], and non-randomized pre–post studies (*n* = 10) [18,21,22,24,25,26,27,30,31,32].

A total of 14,887 participants were included, of whom 94.2% were non-dialysis participants. Intervention duration ranged from 0.5 to 36 months. Across the included studies, mHealth interventions primarily focused on dietary self-monitoring that included nutrient tracking (e.g., phosphorus, potassium, sodium, protein, and fluid intake), nutrition education tailored to individuals receiving dialysis or those with non-dialysis CKD, medication management, and behavioral support strategies designed to improve dietary adherence and self-management.

Sixteen studies (69.6%) utilized mHealth applications specifically developed by the research team [17,19,20,21,22,23,24,28,29,30,31,34,36,37,38,42]. Twelve studies integrated a theory, model, and/or framework to assess the effectiveness of the intervention. These included social cognitive theory (*n* = 4) [17,35,38,42], theory of planned behavior (*n* = 1) [37], models for app development (*n* = 4) [22,28,29,34], or a combination of multiple theories (*n* = 3) [18,20,32]. Eighteen studies (78.3%) incorporated a dietary self-monitoring or tracking component, including food or nutrient logging, meal-photo analysis, nutrient calculation, or monitoring of diet and/or fluid intake. Fifteen studies (65.2%) incorporated educational components, including renal-friendly recipes, medication and dialysis education, and healthy lifestyle guidance. Additionally, 11 studies (47.8%) included behavioral support features such as motivational messaging, reminders, videoconferencing, or direct communication with healthcare providers (Table 1).

Six studies evaluated dietary intake outcomes, including three studies among adults receiving dialysis (*n* = 178) and three among adults with non-dialysis CKD (*n* = 87). For the studies of adults receiving dialysis, protein intake was assessed in three studies, phosphorus intake in three studies, sodium intake in two studies, and overall diet quality in one study. Two of the three studies assessing protein intake reported significant changes (*p* < 0.01), while one of three studies assessing phosphorus intake reported a significant reduction (*p* = 0.003) and one of two studies assessing sodium intake reported a significant reduction (*p* = 0.02) of those in the intervention compared to the control. No significant changes were reported for the remaining dietary outcomes among adults receiving dialysis.

Of the studies of adults with non-dialysis CKD, sodium intake was assessed in three studies, diet quality in two studies, and potassium and protein intake in one study each. Because of variability in the dietary outcomes and statistical metrics reported, most dietary outcomes could not be quantitatively synthesized. The three studies assessing dietary sodium intake reported reductions in sodium intake; however, because these studies used uncontrolled pre–post designs, they were summarized narratively rather than included in the meta-analysis. Among adults with non-dialysis CKD, blood pressure was assessed in four studies [18,30,32,37]. Three pre–post studies [18,30,32] reported limited or no improvements in systolic and/or diastolic blood pressure, whereas the RCT [37] reported no significant between-group differences in blood pressure. Three studies reported serum calcium outcomes [16,23,31]; however, only two provided sufficient data for quantitative synthesis. Given the limited number of eligible studies and the uncertainty associated with estimating between-study heterogeneity, calcium outcomes were summarized narratively rather than pooled.

Sixteen studies assessed at least one self-reported outcome related to dietary self-management, app use, nutrition knowledge, or quality of life. The most frequently assessed outcomes were dietary self-monitoring or self-efficacy (*n* = 6), app satisfaction or acceptability (*n* = 5), nutrition knowledge (*n* = 3), technology readiness (*n* = 3), and quality of life (*n* = 3). Because of variability in the measures used, these outcomes were not quantitatively synthesized. Improvements were reported for nutrition knowledge, dietary self-efficacy, and quality of life. Nutrition knowledge improved significantly in the intervention group compared with the control group in one study (*p* < 0.001), and dietary self-efficacy improved in one study. Two studies reported significant improvements in overall or domain-specific quality of life (*p* < 0.05). Among studies reporting satisfaction with app use, satisfaction was generally high. No significant changes were reported for the remaining self-reported outcomes (Table 1).

### 3.2. Quality and Risk of Bias Results

Risk of bias was assessed using Covidence’s quality assessment templates. Overall, the included studies demonstrated low to moderate risk of bias. Among the randomized controlled trials (RCTs), all studies were judged to have a low risk of bias for incomplete outcome data, selective reporting, and other sources of bias. Four studies were judged to have an unclear risk of bias for blinding of participants and personnel, five for blinding of outcome assessment, and three for allocation concealment due to insufficient reporting of these methodological procedures.

**Figure 2:**
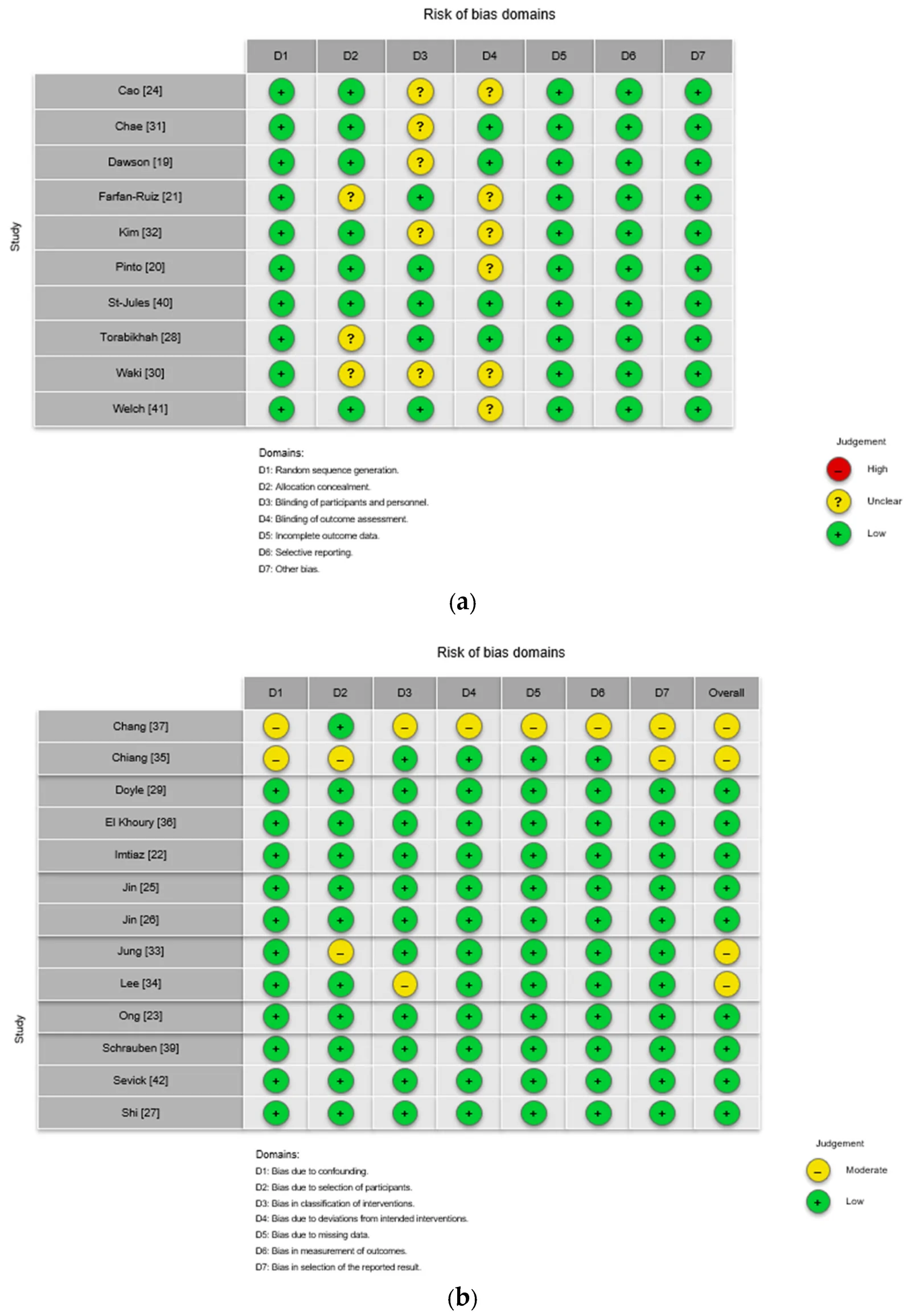
(**a**) Risk-of-bias assessments of included studies. (**a**) Randomized controlled trials assessed using the Cochrane Risk of Bias (RoB) tool [39]. (**b**) Non-randomized studies assessed using the Risk of Bias in Non-randomized Studies of Interventions (ROBINS-I) tool [40]. Visualizations were generated using the robvis package [41].

Non-randomized studies were assessed using the ROBINS-I tool. Ten studies were judged to have a low overall risk of bias, while four studies were rated as having a moderate risk of bias. Moderate ratings were attributable to concerns across domains including confounding, selection of participants, classification of interventions, deviations from intended interventions, outcome measurement, and selection of the reported result. No studies were judged to have a serious or critical risk of bias (Figure 2).

### 3.3. Heterogeneity

Publication bias could not be reliably assessed for most outcomes because of the small number of studies included in each meta-analysis. Heterogeneity varied across outcomes, with substantial heterogeneity observed for several biochemical outcomes. Serum albumin demonstrated particularly high heterogeneity (I^2^ = 8%). Heterogeneity statistics for individual outcomes are presented in the corresponding forest plots.

### 3.4. Meta-Analysis Results

#### 3.4.1. Biochemical Outcomes

No significant differences were observed between intervention and control groups for serum albumin, phosphorus, or potassium concentrations. No significant difference was observed for serum albumin (6 studies, *n* = 476; MD = 0.04 g/dL, 95% CI: −0.21 to 0.28, *p* = 0.72; I^2^ = 85%). Serum phosphorus also showed no significant difference between groups despite substantial heterogeneity across studies (8 studies, *n* = 579; MD = −0.16, 95% CI: −0.33 to 0.00, *p* = 0.06; I^2^ = 78%). Serum potassium concentrations did not significantly differ between groups (5 studies, *n* = 321; MD = −0.35 mmol/L, 95% CI: −1.09 to 0.38, *p* = 0.25; I^2^ = 92%) (Figure S1).

#### 3.4.2. Weight Outcomes

**Figure 3:**
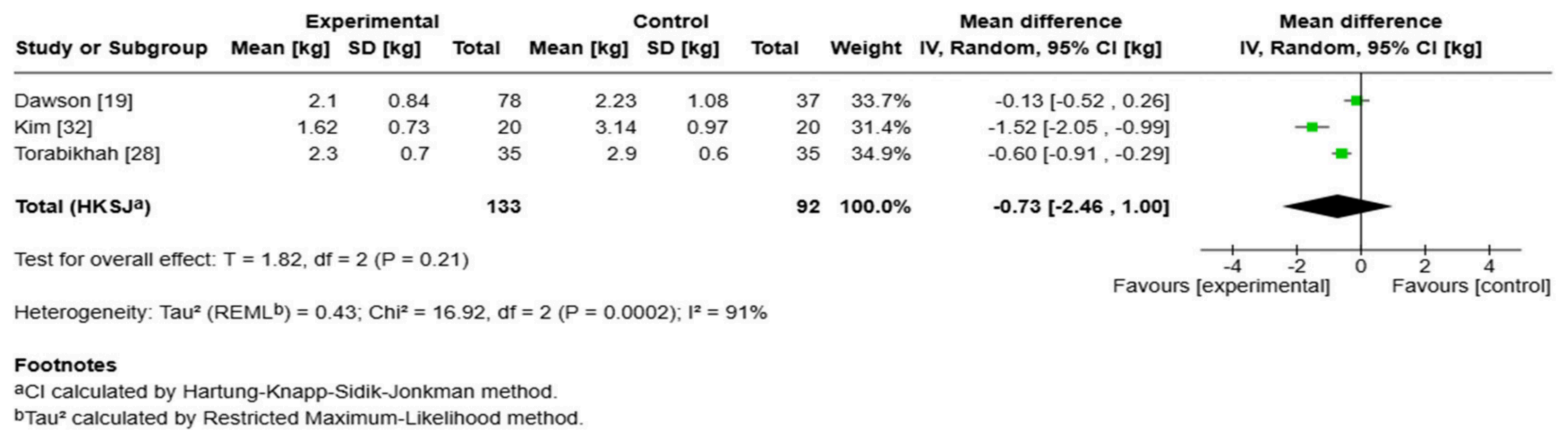
Forest plot of interdialytic weight gain. Effect estimates were pooled using a random-effects model of the interventions among adults undergoing dialysis. Squares represent study-specific effect estimates and horizontal lines represent 95% confidence intervals. Diamonds represent pooled effect estimates. Green squares represent the effect estimates of individual studies, with square size proportional to study weight; horizontal lines indicate 95% confidence intervals. The black diamond represents the pooled effect estimate, with its width indicating the 95% confidence interval.

Three studies involving adults receiving dialysis (*n* = 225) evaluated IDWG. Other studies reporting IDWG were not included in the quantitative synthesis because they lacked a concurrent comparison group or sufficient data to calculate a comparable effect estimate and its variance. The pooled analysis demonstrated no significant differences among the mHealth intervention and the control groups (MD = −0.73 kg, 95% CI −2.46 to 1.00, *p* = 0.21) with substantial heterogeneity across studies (I^2^ = 91%) (Figure 3).

## 4. Discussion

This systematic review and meta-analysis suggest that mHealth-delivered interventions may support selected nutrition and clinical outcomes among adults with CKD; however, effects were not consistent across outcomes or populations. Among adults receiving dialysis, no statistically significant between-group differences were observed in IDWG or the biochemical outcomes evaluated. Among adults with non-dialysis CKD, pre–post studies reported improvements in dietary sodium intake and mixed results in blood pressure. Improvements in nutrition knowledge, dietary self-efficacy, and quality of life were also reported in individual studies. Collectively, these findings suggest that mHealth may be more effective for supporting behaviors directly targeted by the intervention than for producing short-term changes in biochemical outcomes.

Modifying diets for any disease, especially CKD, is a challenge, as multiple factors may compete with adherence, such as culture, preferences, finances, and other co-morbidities [5,6,7]. From this review, there was an overall reduction in dietary sodium intake among individuals with non-dialysis CKD. The reduction in sodium intake observed in these studies may reflect the emphasis placed on sodium reduction within those interventions. For these interventions, not only did participants need to log their dietary intake, but they also incorporated education on reducing sodium intake, encouraged consumption of healthier foods such as fruits and vegetables, and provided dietary tracking with immediate feedback. Jung et al. (2023) [27] illustrated that among 46 participants with non-dialysis CKD (eGFR average 86.5 ±26.5 mL/min/1.73m2), average dietary sodium intake reduced from 2020.9 ± 835.2 mg at baseline to 1456.0 ± 687.3 mg after 6 months. For this intervention, beyond logging meals in an app for 6 months, they were able to correspond with a human coach through the application. The human coaches not only provided feedback but also motivation for daily logging. Even though the other studies included within this systematic review and meta-analysis incorporated some form of counseling with app tracking, there were inconsistencies in diet-related outcomes. This may have been attributed to the type of counseling provided along with the diet app tracking. For example, El Khoury et al.’s study (2021) [22] demonstrated that dietary protein intake and self-knowledge about phosphorus among dialysis participants (*n* = 23) improved, whereas there was no improvement in dietary phosphate consumption, even though the focus of the intervention was on dietary phosphate. For that study, it is not known how the information was communicated and if the focus was specifically on phosphorus or diet information in general. Although sodium and macronutrient information are routinely displayed on Nutrition Facts labels, phosphorus and potassium content may not always be provided. Consequently, individuals with CKD may have difficulty identifying foods containing substantial amounts of these nutrients, which may complicate dietary self-management [27,43,44]. Furthermore, individuals may have limited knowledge regarding the nutrient composition of foods, making it more difficult to identify foods high in nutrients such as sodium, phosphorus, or potassium and to understand how excessive intake may contribute to CKD progression and related complications [45,46,47].

Blood pressure reduction within some studies further supports the potential clinical relevance of sodium reduction achieved through mHealth interventions. Among the studies that evaluated blood pressure, Ong et al. (2016) [30] demonstrated a reduction among the studies included from at home results. The intervention in this study incorporated additional self-management components besides just an app, such as home blood pressure monitoring, symptom assessments, and lab results tracking. Additionally, the intervention integrated developing a personalized feedback loop for patients, and a tiered clinical alert system. This combination of continuous feedback and patient monitoring may explain the blood pressure improvements observed. Schrauben et al. (2022) [32] showed the most consistent improvement across both SBP and DBP and reduction in dietary sodium intake throughout their 12-month intervention among 44 individuals with CKD stages 1–3 compared to the other studies that focused on blood pressure as the outcome. This may have been attributed to the intervention not only including an app that was developed based on two theories—situated learning and control systems—but also integrating tele-counseling, personalized feedback, and motivational interviewing. The dynamic integration of professional dietary support, such as dietitians, along with mHealth technology, may explain the blood pressure and dietary improvements.

Despite improvements in self-management behaviors reported in several studies, these changes did not consistently translate into improvements in biochemical outcomes. Serum phosphorus, potassium, albumin, and calcium are influenced by numerous factors beyond dietary intake, including medication adherence, residual kidney function, dialysis adequacy, inflammation, comorbid conditions, and individual metabolic variability. Consequently, modest dietary improvements achieved through mHealth interventions may not be sufficient to produce measurable changes in laboratory parameters, particularly during short intervention periods. Furthermore, many applications relied on self-reported dietary intake, which may introduce reporting bias and limit the precision of nutrient estimates [48,49]. As a result, improvements in dietary awareness and self-monitoring may occur before detectable changes in biochemical markers become apparent. Furthermore, substantial heterogeneity was observed for some pooled outcomes, which may reflect differences in intervention content and duration, participant characteristics, dialysis modality, and approaches to outcome assessment across studies. Given the limited number of studies available for individual outcomes, additional subgroup or sensitivity analyses to explore potential sources of heterogeneity were not feasible. Therefore, pooled estimates with substantial heterogeneity should be interpreted cautiously.

From a clinical perspective, mHealth may be most useful as a tool to support ongoing nutrition self-management rather than as a standalone intervention. Dietary tracking and self-monitoring were common components of the included interventions, but many studies also incorporated nutrition education, personalized feedback, behavioral support, or communication with healthcare professionals. These findings suggest that mHealth may provide an opportunity to reinforce nutrition recommendations between clinical encounters and facilitate continued engagement with dietary self-management. However, the optimal combination and intensity of technology-based and healthcare professional support remains unclear.

### Limitations

This review has several limitations. Considerable heterogeneity existed across studies in study design, intervention duration, mHealth components, and outcomes assessed, which limited comparisons across interventions. The inclusion of randomized, quasi-experimental, and pre–post studies, along with reliance on self-reported measures for several dietary and behavioral outcomes, may also affect the strength of the findings. Additionally, the distribution of participants across CKD populations was uneven, and several outcomes were specific to either non-dialysis or dialysis populations, limiting generalizability across the CKD continuum. Furthermore, standard deviations for one study were approximated from rounded, model-based confidence intervals, introducing uncertainty into the corresponding pooled estimates.

## 5. Conclusions

mHealth-delivered nutrition interventions show potential for supporting dietary self-management and selected clinical outcomes among adults with CKD, particularly when technology is combined with dietary self-monitoring, personalized feedback, education, or healthcare professional support. However, effects on biochemical outcomes were inconsistent, and substantial heterogeneity in intervention design, study methodology, and outcome assessment limits definitive conclusions. Future adequately powered randomized trials should evaluate standardized behavioral, dietary, and clinical outcomes and determine which components of multicomponent mHealth interventions contribute most to effectiveness.

## Data Availability

The original contributions presented in this study are included in the article/Supplementary Material. Further inquiries can be directed to the corresponding author.

## Supplementary Materials

The following supporting information can be downloaded at: https://www.mdpi.com/article/10.3390/nu18203357/s1, Table S1: Database Search Strategies; Figure S1. Forest plots of biochemical outcomes among adults receiving dialysis. (A) Forest plot of serum albumin concentrations; (B) Forest plot of serum phosphorus concentrations; (C) Forest plot of serum potassium concentrations. Effect estimates were pooled using a random-effects model of the interventions that had individuals on dialysis. Green squares represent study-specific effect estimates and horizontal lines represent 95% confidence intervals. Black diamonds represent pooled effect estimates

## Abbreviations

The following abbreviations are used in this manuscript:

AI	artificial intelligence
CI	confidence interval
CKD	chronic kidney disease
DBP	diastolic blood pressure
eGFR	estimated glomerular filtration rate
MD	mean difference
mHealth	mobile health
PDA	personal digital assistant
PRISMA	Preferred Reporting Items for Systematic Reviews and Meta-Analyses
RCT	randomized controlled trial
RDN	registered dietitian nutritionist
RevMan	Review Manager
RoB	Risk of Bias
ROBINS-I	Risk Of Bias In Non-randomized Studies of Interventions
SBP	systolic blood pressure
SMS	short message service
WHO	World Health Organization
