# Supplementary material for: Effectiveness of mHealth Nutrition Interventions on Nutrition-Related and Clinical Outcomes in Adults with Chronic Kidney Disease: A Systematic Review and Meta-Analysis
Source: Nutrients. 2026 Oct 13;18(20):3357. doi: 10.3390/nu18203357 (PMC13708690; doi:10.3390/nu18203357)
Supplement: Supplementary file 1 [file nutrients-18-03357-s001.zip › nutrients-4591041-supplementary.pdf]

A.

Study or Subgroup	Experimental Mean [g/dL]	Experimental SD [g/dL]	Experimental Total	Control Mean [g/dL]	Control SD [g/dL]	Control Total	Weight	Mean difference IV, Random, 95% CI [g/dL]
Cao [24]	3.4	0.63	70	3.4	0.67	65	15.5%	0.00 [-0.22 , 0.22]
Chae [31]	3.79	0.42	27	3.25	0.56	26	13.7%	0.54 [0.27 , 0.81]
Chiang [35]	3.8	0.3	30	3.9	0.2	30	18.6%	-0.10 [-0.23 , 0.03]
Dawson [19]	3.3	0.44	78	3.3	0.36	37	17.9%	0.00 [-0.15 , 0.15]
Lee [34]	3.58	0.43	21	3.72	0.37	22	14.7%	-0.14 [-0.38 , 0.10]
Torabikhah [28]	3.9	0.2	35	3.9	0.2	35	19.6%	0.00 [-0.09 , 0.09]
**Total (HKSJ[a])**			**261**			**215**	**100.0%**	**0.04 [-0.21 , 0.28]**

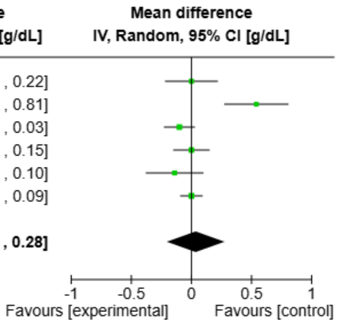

Test for overall effect: T = 0.38, df = 5 (P = 0.72)

Heterogeneity: Tau² (REML[b], 95% CI) = 0.04 [0.01 , 0.35]; Chi² = 19.28, df = 5 (P = 0.002); I² = 85%

Footnotes

[a]CI calculated by Hartung-Knapp-Sidik-Jonkman method.

[b]Tau² calculated by Restricted Maximum-Likelihood method.

B.

Study or Subgroup	Experimental Mean [mmol/L]	Experimental SD [mmol/L]	Experimental Total	Control Mean [mmol/L]	Control SD [mmol/L]	Control Total	Weight	Mean difference IV, Random, 95% CI [mmol/L]
Cao [24]	1.66	0.45	70	1.92	0.39	65	14.5%	-0.26 [-0.40 , -0.12]
Chae [31]	1.85	0.42	27	1.85	0.5	26	11.0%	0.00 [-0.25 , 0.25]
Chiang [35]	1.81	0.45	30	1.87	0.42	30	11.9%	-0.06 [-0.28 , 0.16]
Dawson [19]	1.54	0.51	78	1.61	0.59	37	11.8%	-0.07 [-0.29 , 0.15]
Farfan-Ruiz [21]	1.85	0.44	27	1.96	0.41	36	12.1%	-0.11 [-0.32 , 0.10]
Kim [32]	1.32	0.38	20	1.94	0.3	20	12.1%	-0.62 [-0.83 , -0.41]
Lee [34]	1.77	0.45	21	1.9	0.41	22	10.7%	-0.13 [-0.39 , 0.13]
Torabikhah [28]	1.16	0.19	35	1.2	0.19	35	15.9%	-0.04 [-0.13 , 0.05]
**Total (HKSJ[a])**			**308**			**271**	**100.0%**	**-0.16 [-0.33 , 0.00]**

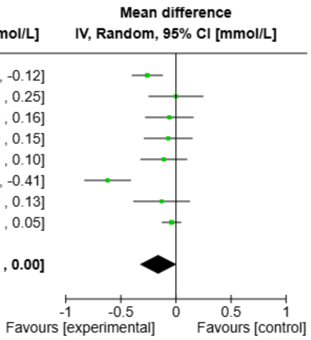

Test for overall effect: T = 2.30, df = 7 (P = 0.06)

Heterogeneity: Tau² (REML[b]) = 0.03; Chi² = 29.39, df = 7 (P = 0.0001); I² = 78%

Footnotes

[a]CI calculated by Hartung-Knapp-Sidik-Jonkman method.

[b]Tau² calculated by Restricted Maximum-Likelihood method.

C.

Study or Subgroup	Experimental Mean [mmol/L]	Experimental SD [mmol/L]	Experimental Total	Control Mean [mmol/L]	Control SD [mmol/L]	Control Total	Weight	Mean difference IV, Random, 95% CI [mmol/L]
Chae [31]	4.15	0.68	27	4.35	0.54	26	20.3%	-0.20 [-0.53 , 0.13]
Dawson [19]	4.8	0.44	78	5.1	0.76	37	20.9%	-0.30 [-0.56 , -0.04]
Kim [32]	4.09	0.43	20	5.44	0.68	20	20.0%	-1.35 [-1.70 , -1.00]
Lee [34]	4.32	0.65	21	4.44	1.09	22	17.9%	-0.12 [-0.65 , 0.41]
Torabikhah [28]	4.6	0.6	35	4.4	0.5	35	20.9%	0.20 [-0.06 , 0.46]
**Total (HKSJ[a])**			**181**			**140**	**100.0%**	**-0.35 [-1.09 , 0.38]**

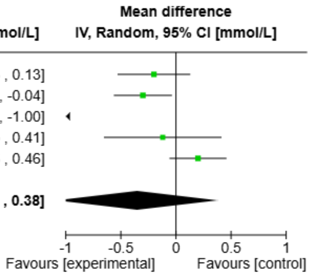

Test for overall effect: T = 1.34, df = 4 (P = 0.25)

Heterogeneity: Tau² (REML[b]) = 0.32; Chi² = 49.17, df = 4 (P < 0.00001); I² = 92%

Footnotes

[a]CI calculated by Hartung-Knapp-Sidik-Jonkman method.

[b]Tau² calculated by Restricted Maximum-Likelihood method.

Figure S1. Forest plots of biochemical outcomes among adults receiving dialysis. (A) Forest plot of serum albumin concentrations; (B) Forest plot of serum phosphorus concentrations; (C) Forest plot of serum potassium concentrations. Effect estimates were pooled using a random-effects model of the interventions that had individuals on dialysis. Green squares represent study-specific effect estimates and horizontal lines represent 95% confidence intervals. Black diamonds represent pooled effect estimates.

Table S1. Database Search Strategies

Database	Search	Search terms	Filters/Limits
PubMed	Queue box #1	(Chronic kidney disease OR kidney disease OR kidney failure OR kidney insufficiency OR kidney function OR kidney dysfunction OR renal disease OR renal failure OR renal insufficiency OR renal function OR renal dysfunction) AND (Applications OR apps OR Smartphones OR wearable devices OR watches OR fitness trackers OR monitoring platforms OR wireless technologies OR scanners OR scales) AND (Phosphorus OR potassium OR sodium OR albumin OR total protein OR dietary habits OR dietary intake)	Text availability: Full text; Article type: Clinical trial and randomized control trial; Age: Adult 19+ years, Young Adult 19–24 years, Adult 19–44 years, Middle Aged+, Aged 45+ years, Middle Aged 45–64 years, Aged 65+ years, 80 and over 80+ years; Language: English; Species: Humans.
	Queue box #2	(Chronic kidney disease OR kidney disease OR kidney failure OR kidney insufficiency) AND (mhealth OR apps OR Smartphones OR wearable devices OR watches OR fitness trackers) AND (Phosphorus OR potassium OR sodium OR albumin)	Text availability: Full text; Article type: Clinical trial and randomized control trial; Age: Adult 19+ years, Young Adult 19–24 years, Adult 19–44 years, Middle Aged+, Aged 45+ years, Middle Aged 45–64 years, Aged 65+ years, 80 and over 80+ years; Language: English; Species: Humans.
	Queue box #3	("chronic kidney disease" OR "kidney disease" OR "kidney failure" OR "kidney insufficiency" OR "kidney function" OR "kidney dysfunction" OR "renal disease" OR "renal failure" OR "renal insufficiency" OR "renal function" OR "renal dysfunction") AND ("mhealth" OR "applications" OR "apps" OR "smartphones" OR "wearable devices" OR "watches" OR "fitness trackers" OR "monitoring platforms" OR "wireless technologies" OR "scanners" OR "scales") AND ("Phosphorus" OR "potassium" OR "sodium" OR "albumin" OR "total protein" OR "dietary habits" OR "dietary intake")	Text availability: Full text; Article type: Clinical trial and randomized control trial; Age: Adult 19+ years, Young Adult 19–24 years, Adult 19–44 years, Middle Aged+, Aged 45+ years, Middle Aged 45–64 years, Aged 65+ years, 80 and over

Database	Search	Search terms	Filters/Limits
			80+ years; Language: English; Species: Humans.
Cochrane	Search #1	"Chronic kidney disease" OR "kidney disease" OR "kidney failure" OR "kidney insufficiency" OR "kidney function" OR "kidney dysfunction" OR "renal disease" OR "renal failure" OR "renal insufficiency" OR "renal function" OR "renal dysfunction"<br>AND<br>"mHealth" OR "Applications" OR "apps" OR "smartphones" OR "wearable devices" OR "watches" OR "fitness trackers" OR "monitoring platforms" OR "wireless technologies" OR "scanners" OR "scales"<br>AND<br>"phosphorus" OR "potassium" OR "sodium" OR "albumin" OR "total protein" OR "dietary habits" OR "dietary intake"<br>AND<br>"adult" OR "adults"	None reported.
	Search #2	"Chronic kidney disease" OR "kidney disease" OR "kidney failure" OR "kidney insufficiency" OR "kidney function" OR "kidney dysfunction" OR "renal disease" OR "renal failure" OR "renal insufficiency" OR "renal function" OR "renal dysfunction"<br>AND<br>"mHealth" OR "Applications" OR "apps" OR "smartphones" OR "wearable devices" OR "watches" OR "fitness trackers" OR "monitoring platforms" OR "wireless technologies" OR "scanners" OR "scales"<br>AND<br>"phosphorus" OR "potassium" OR "sodium" OR "albumin" OR "total protein" OR "dietary habits" OR "dietary intake"<br>AND<br>"adult" OR "adults" OR "young adult" OR "middle aged" OR "aged"	None reported.
Embase	Search #1	('Chronic kidney failure' OR 'kidney disease' OR 'kidney failure') AND ('application' OR 'app' OR 'smartphone' or 'wearable device') AND ('phosphorus' OR 'potassium' OR 'sodium' OR 'albumin')	Study types: Human; Controlled; Major clinical study; Randomized control trial; Clinical trial; Prospective study; Pilot study; Clinical controlled study. Publication type: Article; Clinical trail. Ages: Adults; Young adults; Middle aged; Aged.

Database	Search	Search terms	Filters/Limits
	Search #2	('chronic kidney failure' OR 'kidney disease' OR 'kidney failure' OR 'kidney function' OR 'kidney dysfunction') AND ('app' OR 'application' OR 'smartphone' OR 'wearable device' OR 'watch' OR 'fitness tracker' OR 'scales' OR 'scanner') AND ('phosphorus' OR 'potassium' OR 'albumin' OR 'protein' OR 'dietary habit')	Study types: Human; Controlled; Major clinical study; Randomized control trial; Clinical trial; Prospective study; Pilot study; Clinical controlled study. Publication type: Article; Clinical trail. Ages: Adults; Young adults; Middle aged; Aged.
	Search #3	('kidney disease' OR 'renal disease' OR 'renal failure' OR 'renal insufficiency' OR 'renal function') AND ('app' OR 'fitness tracker' OR 'monitoring platform' OR 'wearable device') AND ('dietary intake' OR 'total protein' OR 'sodium' OR 'phosphorus')	Study types: Human; Controlled; Major clinical study; Randomized control trial; Clinical trial; Prospective study; Pilot study; Clinical controlled study. Publication type: Article; Clinical trail. Ages: Adults; Young adults; Middle aged; Aged.
	Search #4	('kidney disease' OR 'kidney failure' OR 'kidney function' OR 'renal disease' OR 'renal insufficiency') AND ('mhealth' OR 'application' OR 'app' OR 'smartphone' OR 'wearable device' OR 'wireless communication') AND ('phosphorus' OR 'potassium' OR 'sodium' OR 'albumin' OR 'electrolyte')	Study types: Human; Controlled; Major clinical study; Randomized control trial; Clinical trial; Prospective study; Pilot study; Clinical controlled study. Publication type: Article; Clinical trail. Ages: Adults; Young adults; Middle aged; Aged.
	Search #5	('chronic kidney disease' OR 'kidney disease' OR 'kidney failure' OR 'kidney insufficiency' OR 'kidney function' OR 'kidney dysfunction' OR 'renal disease' OR 'renal failure' OR 'renal insufficiency' OR 'renal function' OR 'renal dysfunction') AND ('app' OR 'application' OR 'smartphone' OR 'wearable device' OR 'watch' OR 'fitness tracker' OR 'monitoring platform' OR	Study types: Human; Controlled; Major clinical study; Randomized control trial; Clinical trial; Prospective study; Pilot

Database	Search	Search terms	Filters/Limits
		'wireless technology' OR 'scanner' OR 'scales' OR 'phosphorus' OR 'potassium' OR 'sodium' OR 'albumin' OR 'total protein' OR 'dietary habit' OR 'dietary intake')	study; Clinical controlled study. Publication type: Article; Clinical trail. Ages: Adults; Young adults; Middle aged; Aged.
	Search #6	('kidney disease' OR 'kidney failure' OR 'chronic kidney failure') AND 'adult' AND 'mhealth' AND ('electrolyte' OR 'sodium' OR 'potassium' OR 'phosphorus')	Study types: Human; Controlled; Major clinical study; Randomized control trial; Clinical trial; Prospective study; Pilot study; Clinical controlled study. Publication type: Article; Clinical trail. Ages: Adults; Young adults; Middle aged; Aged.
SciELO	Database search	CKD/renal terms (e.g., chronic kidney disease, kidney disease/failure/insufficiency, renal disease/failure/insufficiency) AND mHealth/digital technology terms (e.g., mHealth, application/app, smartphone, wearable device, fitness tracker, monitoring platform) AND nutrition-related terms/outcomes (e.g., phosphorus, potassium, sodium, albumin/protein, dietary habits/intake). Exact database-specific search syntax was not retained in the search documentation.	No filters/limits applied
Scopus	Search #1	(‘Chronic kidney failure’ OR ‘kidney disease’ OR ‘kidney failure’) AND (‘application’ OR ‘app’ OR ‘smartphone’ or ‘wearable device’) AND (‘phosphorus’ OR ‘potassium’ OR ‘sodium’ OR ‘albumin’)	Study types: Human; Controlled; Major clinical study; Randomized control trial; Clinical trial; Prospective study; Pilot study; Clinical controlled study. Publication type: Article; Clinical trail. Ages: Adults; Young adults; Middle aged; Aged.
	Search #2	('chronic kidney failure' OR 'kidney disease' OR 'kidney failure' OR 'kidney function' OR 'kidney dysfunction') AND ('app' OR 'application' OR 'smartphone' OR 'wearable device' OR 'watch' OR 'fitness tracker' OR 'scales' OR 'scanner') AND ('phosphorus' OR 'potassium' OR 'albumin' OR 'protein' OR 'dietary habit')	Study types: Human; Controlled; Major clinical study; Randomized control trial; Clinical trial; Prospective study; Pilot

Database	Search	Search terms	Filters/Limits
			study; Clinical controlled study. Publication type: Article; Clinical trail. Ages: Adults; Young adults; Middle aged; Aged.
	Search #3	('kidney disease' OR 'renal disease' OR 'renal failure' OR 'renal insufficiency' OR 'renal function') AND ('app' OR 'fitness tracker' OR 'monitoring platform' OR 'wearable device') AND ('dietary intake' OR 'total protein' OR 'sodium' OR 'phosphorus')	Study types: Human; Controlled; Major clinical study; Randomized control trial; Clinical trial; Prospective study; Pilot study; Clinical controlled study. Publication type: Article; Clinical trail. Ages: Adults; Young adults; Middle aged; Aged.
	Search #4	('kidney disease' OR 'kidney failure' OR 'kidney function' OR 'renal disease' OR 'renal insufficiency') AND ('mhealth' OR 'application' OR 'app' OR 'smartphone' OR 'wearable device' OR 'wireless communication') AND ('phosphorus' OR 'potassium' OR 'sodium' OR 'albumin' OR 'electrolyte')	Study types: Human; Controlled; Major clinical study; Randomized control trial; Clinical trial; Prospective study; Pilot study; Clinical controlled study. Publication type: Article; Clinical trail. Ages: Adults; Young adults; Middle aged; Aged.

Note. Search strings, filters, and dates for PubMed, Cochrane, and Embase are reproduced from the contemporaneous search documentation and have not been retrospectively altered. SciELO and Scopus were searched using combinations of the same three core concepts (CKD/renal disease, mHealth/digital technology, and nutrition-related terms/outcomes); however, the exact database-specific syntax and filters were not retained. These fields are therefore reported as not recorded rather than reconstructed retrospectively.
